# Few-shot network intrusion detection method based on multi-domain fusion and cross-attention

**DOI:** 10.1371/journal.pone.0327161

**Published:** 2025-07-02

**Authors:** Congyuan Xu, Donghui Li, Zihao Liu, Jun Yang, Qinfeng Shen, Ningbing Tong

**Affiliations:** 1 School of Electrical and Information Engineering, Tianjin University, Tianjin, China; 2 College of Artificial Intelligence, Jiaxing University, Jiaxing, Zhejiang, China; 3 Zhejiang Hongxiang Food Co., Ltd., Jiaxing, Zhejiang, China; University of Alaska Anchorage, UNITED STATES OF AMERICA

## Abstract

Deep learning methods have achieved remarkable progress in network intrusion detection. However, their performance often deteriorates significantly in real-world scenarios characterized by limited attack samples and substantial domain shifts. To address this challenge, we propose a novel few-shot intrusion detection method that integrates multi-domain feature fusion with a bidirectional cross-attention mechanism. Specifically, the method adopts a dual-branch feature extractor to jointly capture spatial and frequency domain characteristics of network traffic. The frequency domain features are obtained via two-dimensional discrete cosine transform (2D-DCT), which helps to highlight the spectral structure and improve feature discriminability. To bridge the semantic gap between support and query samples under few-shot conditions, we design a dual-domain bidirectional cross-attention module that enables deep, task-specific alignment across spatial and frequency domains. Additionally, we introduce a hierarchical feature encoding module based on a modified Mamba architecture, which leverages state space modeling to capture long-range dependencies and temporal patterns in traffic sequences. Extensive experiments on two benchmark datasets, CICIDS2017 and CICIDS2018, demonstrate that the proposed method achieves accuracy of 99.03% and 98.64% under the 10-shot setting, outperforming state-of-the-art methods. Moreover, the method exhibits strong cross-domain generalization, achieving over 95.13% accuracy in cross-domain scenarios, thereby proving its robustness and practical applicability in real-world, dynamic network environments.

## 1 Introduction

With the rapid advancement of digital technologies, network infrastructures have become increasingly complex, and cyberattacks are evolving towards higher frequency, diversity, and intelligence. This poses severe challenges to the security of information systems. As a critical component of cybersecurity defense, intrusion detection systems (IDS) aim to identify potential malicious behaviors and abnormal traffic in real time, thereby preventing further attack propagation [1]. Traditional rule-based IDS methods, however, often struggle to cope with novel or mutated attacks due to their limited generalization capability, rendering them insufficient for the dynamic requirements of modern network environments [2].

In recent years, deep learning has been widely applied to intrusion detection tasks owing to its powerful representation learning capabilities, significantly improving the identification accuracy of complex attack behaviors [3]. Nevertheless, most deep learning-based methods require a large volume of labeled samples and are highly sensitive to the distribution consistency between training and testing datasets. In practical scenarios, these methods face two major challenges: (1) attack samples are inherently scarce; and (2) there exist significant domain shifts across different network environments, leading to degraded model robustness and adaptability [4]. Therefore, it remains a key research problem to develop an intrusion detection method that can maintain high discriminative power under limited samples and varying data distributions.

Leveraging the strong nonlinear modeling capacity of deep learning, remarkable breakthroughs have been achieved in computer vision [5], natural language processing [6], and speech recognition [7], and similar methods have been successfully extended to intrusion detection. By constructing deep neural networks, systems can automatically extract multi-level features from raw network traffic and uncover latent attack patterns, thus enabling efficient recognition of unknown threats [8]. Compared with traditional detection methods that rely heavily on handcrafted features and rule matching, deep learning-based approaches offer better adaptability and generalization, achieving higher accuracy and robustness in complex attack scenarios. However, their dependence on large-scale labeled data makes them susceptible to performance bottlenecks when facing high data acquisition costs or sparse attack samples. In real-world environments, attack behaviors are diverse and evolve rapidly, leading to very limited or entirely unseen samples of new attack types. Meanwhile, due to privacy concerns, compliance requirements, and security restrictions, constructing comprehensive labeled datasets is often costly and time-consuming [9].

To address this issue, few-shot learning (FSL) offers a promising paradigm for tackling the data scarcity problem in intrusion detection. FSL aims to train models that can generalize to new tasks with only a few labeled examples by simulating the learning process during training [10]. By introducing the *support set–query set* task formulation, FSL enables the model to learn how to adapt quickly to novel classes during inference. Recent research has explored applying few-shot learning to intrusion detection, utilizing metric learning, meta-learning, or contrastive learning frameworks. However, existing methods often rely on single-domain feature modeling (e.g., spatial or statistical features), which limits their ability to capture the rich heterogeneous structure embedded in network traffic. Moreover, similarity calculation between support and query samples is typically performed in shallow feature spaces, lacking deep semantic alignment or cross-domain adaptability.

We propose a novel few-shot intrusion detection framework based on multi-domain feature fusion and cross-attention mechanisms. The proposed method jointly models the spatial and frequency domain features of network traffic and introduces bidirectional cross-attention to enhance dynamic alignment between support and query sets. This enables robust intrusion detection even under extreme data scarcity.

The main contributions of this paper are summarized as follows:

1. A multi-domain feature fusion framework is proposed for few-shot network intrusion detection, incorporating both spatial and frequency domain representations. The use of two-dimensional discrete cosine transform enhances the separability of attack patterns by revealing the spectral structure of traffic samples.
2. A dual-domain bidirectional cross-attention mechanism is designed to construct attention between support and query sets in both spatial and frequency domains, improving feature matching robustness and mitigating instability under few-shot conditions.
3. A hierarchical feature encoding module based on state space modeling is introduced, utilizing an improved Mamba network to extract structured features from both domains and capture long-range dependencies in traffic sequences, thereby enhancing encoding depth and sequence modeling capacity.
4. Extensive experiments on CICIDS2017 and CICIDS2018 demonstrate that the proposed method outperforms existing methods under classic *K*-shot settings. Furthermore, cross-dataset generalization experiments validate its strong adaptability and practical value under distribution shifts.

The remainder of this paper is organized as follows. Sect 2 reviews related work. Sect 3 details the proposed method and key components. Sect 4 presents the experimental setup, results, and detailed analysis. Sect 5 concludes the paper and discusses future research directions.

## 2 Related work

Early network intrusion detection systems (NIDS) primarily relied on handcrafted rules and domain expertise to construct detection models. Common approaches include signature-based detection and anomaly-based detection. Signature-based methods identify malicious behaviors by matching traffic patterns against known attack signatures, offering high efficiency and low false positive rates. However, they fail to detect novel or mutated attacks. Anomaly-based methods, on the other hand, learn the distribution of normal traffic and flag deviations as potential threats, which provides better generalization to unknown attacks but often suffers from high false alarm rates due to noise sensitivity.

In addition to rule-based methods, classical machine learning algorithms have been employed for intrusion detection, such as *k*-nearest neighbors (*k*NN) [11], support vector machines (SVM) [12], and decision trees (DT) [13]. While these approaches improve detection accuracy to some extent, they still rely heavily on feature engineering and often struggle to maintain performance in complex attack scenarios.

With the advancement of deep learning, researchers have begun exploring its application in cybersecurity, inspired by its success in image recognition, speech processing, and other domains. Models such as convolutional neural networks (CNN) [14–16] and recurrent neural networks (RNN) [17–19] have been adopted to model network traffic data. These methods automatically learn hierarchical representations from raw input data, significantly enhancing the ability to detect complex or previously unseen attack patterns. For instance, CNNs excel at capturing local spatial features, making them suitable for visual traffic representations, while RNNs or LSTMs are more adept at modeling sequential dependencies in traffic flows. Despite achieving strong results on benchmark datasets, deep learning models still face two major limitations: (1) a strong dependency on large volumes of labeled data, which restricts their deployment in low-resource scenarios; and (2) poor generalization when faced with distribution shifts between training and testing data, undermining their robustness in real-world environments.

To alleviate the dependency on large-scale labeled datasets, few-shot learning (FSL) has gained significant attention in recent years, particularly in fields like computer vision and natural language processing. FSL aims to mimic the human ability to learn new concepts from a handful of examples by training models that can quickly adapt to novel tasks [20]. FSL approaches generally fall into three categories: metric-based methods [21–24], which learn to measure similarities between samples in an embedding space; optimization-based meta-learning methods [25–28], which train models to rapidly adapt to new tasks with few gradient updates; and hybrid methods, which integrate traditional machine learning components into neural networks to improve few-shot generalization [29].

Inspired by these advances, recent studies have begun incorporating few-shot learning into network intrusion detection, resulting in the emergence of FSL-based IDS. Typical strategies involve applying siamese or prototypical networks to extract and compare traffic features within a few-shot classification framework. For instance, CNNs have been used to encode traffic flows into image representations, followed by similarity-based classification [30,31]; meta-learning methods such as MAML have been adopted to improve adaptation across attack categories [32–34]; and incremental learning techniques have also been explored to support evolving attack scenarios [35].

However, most existing methods remain at an early stage and exhibit several key limitations:

- **Limited feature modeling with insufficient domain fusion.** Most methods focus solely on spatial or statistical domain features, overlooking the potential of frequency-domain representations. Frequency features can reveal important patterns such as periodicity, repetition, and local disturbances in traffic, which are crucial for identifying stealthy or subtle attack behaviors.
- **Shallow support-query matching mechanisms.** Many FSL approaches rely on fixed distance metrics (e.g., Euclidean or cosine distance) to compare samples, which fail to capture complex cross-task semantic relationships. Matching is typically performed in shallow feature spaces without contextual enhancement, making the models vulnerable to noise and irrelevant information.
- **Shallow feature encoders lacking temporal modeling.** Network traffic inherently exhibits strong temporal dependencies, particularly for flow-based attacks like DDoS or botnet communication. Most existing methods use shallow CNNs or basic attention modules, which are insufficient for modeling long-term dependencies across time steps, limiting their ability to capture behavioral patterns in sequence data.

In summary, current few-shot intrusion detection methods face notable limitations in three key aspects: multi-domain feature modeling, deep semantic alignment, and long-range sequence modeling. Addressing these gaps requires enhanced fusion architectures, attention mechanisms capable of deeper interaction, and sequence encoders that can capture both local and global dependencies in network traffic data.

## 3 Methodology

### 3.1 Sample definition

**Fig 1:**
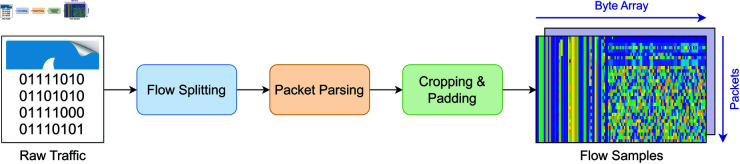
Illustration of converting network traffic into sample matrices.

To enable deep learning models to effectively handle few-shot network intrusion detection tasks, raw network traffic must first be transformed into a standardized input format. As illustrated in Fig 1, the preprocessing pipeline consists of three key steps: flow splitting, packet parsing, and cropping & padding.

**(1) Flow Splitting.** Raw network traffic is typically captured in formats such as PCAP files or live packet captures, where each packet contains header and payload information. In this step, the captured traffic is split into independent flows based on five-tuple attributes: source IP address, source port, destination IP address, destination port, and protocol. Each resulting flow corresponds to a session with shared communication characteristics, facilitating subsequent analysis.

**(2) Packet Parsing.** For each flow, packet contents are extracted, including headers and payloads. To prevent information leakage, IP addresses are masked to eliminate potential class-related identifiers. Other header fields are retained to preserve behavioral features relevant to network activity.

**(3) Cropping and Padding.** Since flows and packets vary significantly in length, payload data is normalized to a fixed size to satisfy neural network input constraints. Specifically, two parameters are defined:

- $n_{\text{packet}}$: Maximum number of packets per flow
- $n_{\text{byte}}$: Fixed number of bytes per packet

If a flow contains more than $n_{\text{packet}}$ packets, only the first $n_{\text{packet}}$ are retained. Otherwise, zero-padding is applied. Similarly, each packet’s payload is cropped or padded to length $n_{\text{byte}}$.

After processing, each network flow is converted into a two-dimensional traffic matrix, where the height corresponds to the number of packets ($n_{\text{packet}}$), and the width corresponds to the payload length ($n_{\text{byte}}$). Each row represents the byte-level content of one packet. This matrix formulation transforms the temporal structure of network traffic into a spatial representation, from which both spatial and implicit temporal features can be extracted by subsequent modules.

### 3.2 Task formulation

Traditional intrusion detection models based on deep learning require extensive labeled datasets to achieve good generalization. However, in practical environments, data labeling is costly, attack samples are sparse, and privacy constraints often hinder dataset construction. To address these challenges, we define intrusion detection as a few-shot classification problem, where each task consists of a *support set* and a *query set*.

Each task follows an *N*-way *K*-shot protocol: the support set contains *K* labeled samples from each of *N* classes, while the query set includes unlabeled samples that must be classified using information from the support set.

Formally, a few-shot task is composed of:

**(1) Support set:** $S=\{(x_{i},y_{i}){\}}_{i=1}^{N\times K}$, where *x*_*i*_ is a preprocessed traffic sample, and $y_{i}\in \{1,\ldots ,N\}$ is the corresponding label. There are *N* classes with *K* samples per class.

**(2) Query set:** $Q=\{x_{j}{\}}_{j=1}^{N_{Q}}$, where *x*_*j*_ denotes the unlabeled traffic samples to be classified, and *N*_*Q*_ is the number of query samples.

The objective is to learn a task-specific classifier $f_{\theta }(x_{q},S)$ that predicts the class of a query sample *x*_*q*_ using the support set *S*:

$${\hat{y}}_{q}=\arg\max_{y\in \{1,\ldots ,N\}}P(y|x_{q},𝒮,\theta )$$ (1)

**Fig 2:**
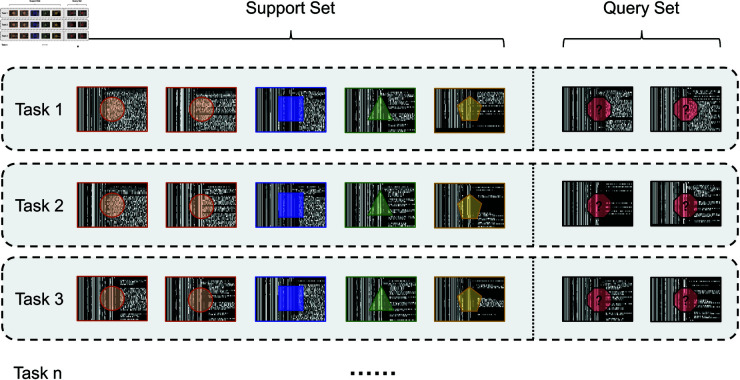
Few-shot task formulation in intrusion detection.

As shown in Fig 2, the few-shot intrusion detection task simulates a realistic scenario in which only limited labeled samples are available for a new or rare attack type. The model must learn to adapt and perform accurate classification using minimal supervision, thus improving generalization under few-shot conditions.

### 3.3 Overall framework

**Fig 3:**
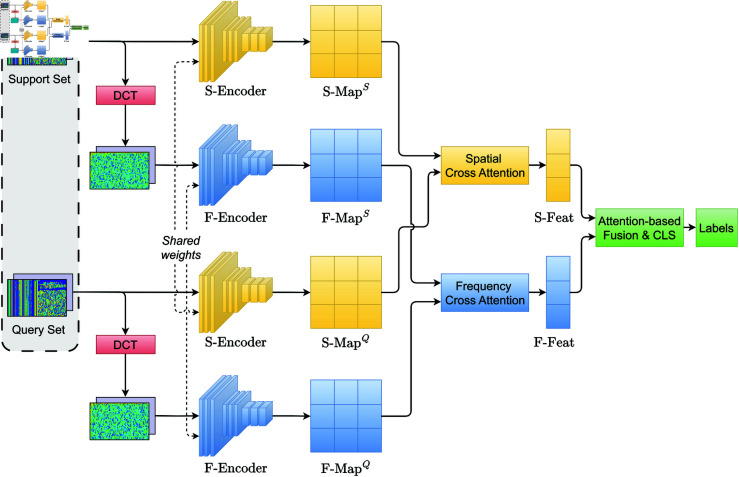
Overall architecture of the proposed few-shot intrusion detection method.

To address the challenges of few-shot learning in network intrusion detection, we propose a framework that integrates multi-domain feature modeling and a bidirectional cross-attention mechanism. As illustrated in Fig 3, the proposed method consists of five modules: preprocessing, frequency domain transformation, feature encoding, feature matching, and feature fusion with classification.

**(1) Preprocessing.** Each detection task contains a support set and a query set. All samples are first normalized to ensure consistent input distributions across the model. This preprocessing step standardizes the inputs, laying the foundation for reliable feature extraction and comparison.

**(2) Frequency Domain Transformation.** Normalized traffic samples are further transformed into the frequency domain using the two-dimensional discrete cosine transform. This operation effectively decomposes the spectral components of network traffic, exposing differences between normal and attack patterns in the frequency domain. After this step, each sample has both spatial and frequency representations, which are used in subsequent modules for joint modeling.

**(3) Feature Encoding.** Two independent encoders are used to extract features from the spatial and frequency domains. The spatial encoder (S-Encoder) processes the original traffic matrix and produces a spatial feature map (S-Map), while the frequency encoder (F-Encoder) operates on the DCT-transformed input and outputs a frequency feature map (F-Map). Both encoders share the same architecture to ensure consistency, and the support/query sets are processed through shared weights.

**(4) Feature Matching.** To compare support and query features, we employ a bidirectional cross-attention mechanism in both the spatial and frequency domains. The spatial cross-attention module computes attention between support and query samples in the spatial feature space, generating the spatial comparison features (S-Feat). Similarly, the frequency cross-attention module computes attention in the frequency space, yielding the frequency comparison features (F-Feat). These mechanisms enhance fine-grained alignment across samples.

**(5) Feature Fusion and Classification.** The resulting S-Feat and F-Feat are fused via an attention-based fusion module, which adaptively integrates multi-domain features. The fused representation is then passed to the classification module (CLS), implemented as a multi-layer perceptron (MLP), to perform final intrusion classification and output predicted labels.

The proposed framework fully leverages spatial, temporal, and frequency-domain features of network traffic. By incorporating bidirectional cross-domain attention, it enhances the mutual alignment between support and query sets, improving detection performance under few-shot conditions.

### 3.4 Frequency domain transformation

In network intrusion detection, traffic samples contain not only spatial and temporal patterns but also critical information in the frequency domain. To exploit such frequency-domain features, we apply the two-dimensional discrete cosine transform (2D-DCT) to map traffic samples from the spatial domain into the frequency domain, thereby enabling the extraction of more discriminative representations.

Given a traffic sample represented as a grayscale image of size H×W, where *x*(*i*,*j*) denotes the signal intensity at position (*i*,*j*), the 2D-DCT is mathematically defined as:

$$F(u,v)=\alpha (u)\alpha (v)\sum_{i=0}^{H-1}\sum_{j=0}^{W-1}x(i,j)\cos\left[\frac{(2i+1)u\pi }{2H}\right]\cos\left[\frac{(2j+1)v\pi }{2W}\right]$$ (2)

where *F*(*u*,*v*) is the DCT coefficient at frequency indices (*u*,*v*), representing the energy distribution in the frequency domain. The normalization factors α(u) and α(v) are defined as:

$$\alpha (u)=\left\{ \begin{array}{cc} \sqrt{\frac{1}{H}}, & \text{if }u=0\\ \sqrt{\frac{2}{H}}, & \text{if }u>0 \end{array}\right.,\;\alpha (v)=\left\{ \begin{array}{cc} \sqrt{\frac{1}{W}}, & \text{if }v=0\\ \sqrt{\frac{2}{W}}, & \text{if }v>0 \end{array}\right.$$ (3)

The resulting frequency spectrum reveals the energy composition across different frequency bands. Low-frequency components (i.e., small *u* and *v*) capture the global structure and background trends of network traffic, while high-frequency components (i.e., large *u* and *v*) represent rapid changes, fine-grained variations, or noise—often crucial for identifying subtle or stealthy attacks.

To illustrate the invertibility of DCT, the inverse discrete cosine transform (IDCT) can be used to reconstruct the original input:

$$x(i,j)=\sum_{u=0}^{H-1}\sum_{v=0}^{W-1}\alpha (u)\alpha (v)F(u,v)\cos\left[\frac{(2i+1)u\pi }{2H}\right]\cos\left[\frac{(2j+1)v\pi }{2W}\right]$$ (4)

This property indicates that DCT preserves essential information while emphasizing the frequency structure. In our detection framework, only the forward DCT is applied during training and inference to enhance feature separability, while IDCT is useful for visualizing or analyzing traffic in the spatial domain when necessary.

By transforming network traffic into the frequency domain, we introduce a complementary perspective to traditional spatial modeling. The frequency domain representation enhances the ability to distinguish between benign and malicious traffic, especially in cases where spatial features alone are insufficient.

### 3.5 Feature encoding

**Fig 4:**
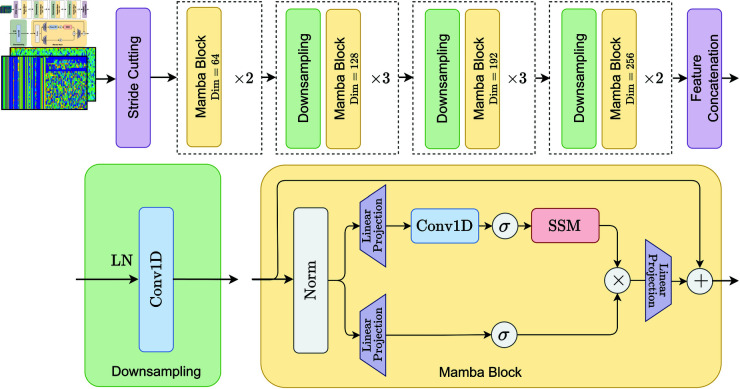
Architecture of the feature encoding module based on Mamba.

To extract expressive representations from both spatial and frequency-domain traffic samples, we design a feature encoding module based on an improved Mamba architecture, as shown in Fig 4. This module includes two structurally identical yet input-specific encoders: the spatial encoder (S-Encoder) and the frequency encoder (F-Encoder). The S-Encoder directly processes raw traffic matrices, while the F-Encoder operates on DCT-transformed frequency-domain inputs. Although their inputs differ in domain, both branches share the same network structure to ensure consistency in representational capacity.

The overall module comprises four main stages: *stride-based partitioning*, *downsampling*, *Mamba-based feature extraction*, and *feature concatenation*.

**(1) Stride-Based Partitioning.** Traffic samples, represented as 2D matrices, are first partitioned into fixed-length sequences using a sliding window with predefined stride. This conversion prepares the data for sequential processing and helps extract localized correlations across the input.

**(2) Downsampling.** To reduce the input sequence length and computational cost, each segment undergoes a combination of layer normalization (LN) and one-dimensional convolution (Conv1D). LN stabilizes the training process by normalizing feature distributions, while Conv1D captures local dependencies and reduces dimensionality.

**(3) Mamba-Based Feature Extraction.** The core of the encoding module is a Mamba block that integrates both nonlinear projection and state space modeling [36]. The Mamba block includes the following key steps:

1. *Normalization and Dual-Channel Projection:* The input is first normalized, followed by two independent linear projections with nonlinear activation functions.
2. *State Space Modeling (SSM):* One branch further processes the features through Conv1D and a continuous-time state space model, described by the following ordinary differential equations (ODEs):
  $$\frac{d}{dt}𝐱(t)=𝐀𝐱(t)+𝐁u(t),\;y(t)=𝐂𝐱(t)+𝐃u(t)$$ (5)
  where $𝐱(t)\in {\mathbb{R}}^{N}$ is the hidden state, *u*(*t*) is the input, and *y*(*t*) is the output. 𝐀,𝐁,𝐂,𝐃 are trainable parameters of appropriate dimensions.
3. *Discretization:* The continuous model is discretized as:
  $${𝐱}_{k+1}={𝐀}_{d}{𝐱}_{k}+{𝐁}_{d}u_{k},\;y_{k}=𝐂{𝐱}_{k}+𝐃u_{k}$$ (6)
  with transition matrices:
  $${𝐀}_{d}=e^{𝐀\Delta t},\;{𝐁}_{d}=\left(\int_{0}^{\Delta t}e^{𝐀\tau }d\tau \right)𝐁$$ (7)
  For simplicity and efficiency, our implementation omits the direct feedthrough term *Du*_*k*_ and uses the reduced form:
  $${𝐱}_{k}={𝐀}_{d}{𝐱}_{k-1}+{𝐁}_{d}u_{k},\;y_{k}=𝐂{𝐱}_{k}$$ (8)
4. *Nonlinear Activation and Fusion:* A GeLU activation function [37] is applied to *y*_*k*_ to introduce nonlinearity:
  $$z_{k}=\text{GeLU}(y_{k})$$ (9)
  This output is then element-wise multiplied with the projection result from the other channel, and fused through a final linear layer to generate the encoded feature.

**(4) Feature Concatenation.** To capture multi-scale semantics, we stack multiple Mamba blocks, interleaved with downsampling operations. The feature dimensionality progressively increases across layers (64 → 128 → 192 → 256). Finally, all intermediate representations are concatenated to form the final hierarchical feature vector.

To enhance feature representation capability, the proposed encoding module integrates an improved Mamba-based architecture with a hierarchical feature extraction strategy, enabling deep modeling of both spatial and frequency domain information. Compared with conventional CNN or Transformer architectures, our module introduces three major innovations:

First, the stride-based partitioning mechanism divides the input traffic matrix into temporal sub-sequences using a sliding window. This allows the model to capture fine-grained local dependencies at early stages, which is especially useful for modeling subtle variations between network packets.

Second, the multi-layered Mamba blocks with downsampling gradually reduce sequence length and expand feature dimensionality. This hierarchical structure enables the model to progressively extract high-level semantic patterns from local to global scales. Thanks to the state space modeling capability of Mamba, the module effectively captures long-range dependencies even in long and noisy traffic sequences, which is an area where self-attention mechanisms typically face scalability and generalization challenges.

Third, the dual-branch encoder structure (S-Encoder and F-Encoder) processes spatial and frequency domain inputs independently using the same architecture. This enables the model to extract complementary representations: spatial features capture structural behavior in packet sequences, while frequency features emphasize periodicity, burst patterns, and other spectral characteristics that may be difficult to detect in the spatial domain alone.

As a result, the enhanced encoding module not only achieves comprehensive modeling of short-term and long-term patterns but also provides robust, domain-aware feature representations that significantly improve support-query alignment in downstream cross-attention-based matching and classification.

### 3.6 Feature matching

**Fig 5:**
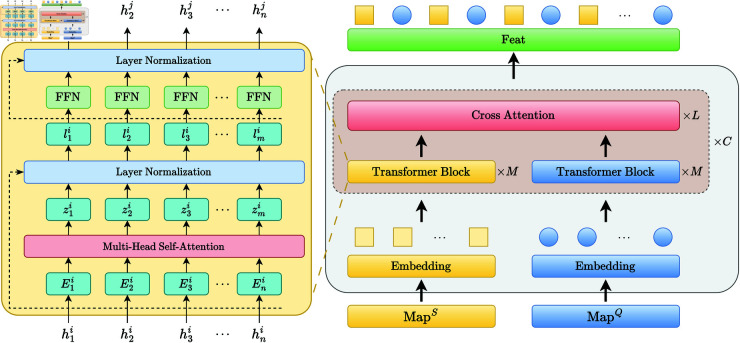
Architecture of the feature matching module with cross-attention.

To enable accurate comparison between support and query samples, we design a bidirectional cross-attention mechanism that operates in both spatial and frequency domains. The feature matching module consists of two main components: a Transformer-based intra-feature encoder and a dual-domain bidirectional cross-attention module, as illustrated in Fig 5.

**1) Transformer-Based Feature Refinement.** Before performing cross-attention, we apply Transformer layers to refine individual features and capture long-range dependencies within each sample. The input features from the support and query sets, denoted as $X=\{x_{1},x_{2},...,x_{L}\}\in {\mathbb{R}}^{L\times d}$, are first embedded into tokens:

$$T=\text{Embed}(X)\in {\mathbb{R}}^{L\times d}$$ (10)

The multi-head self-attention (MHSA) mechanism computes the internal relationships among tokens by projecting them into queries (*Q*), keys (*K*), and values (*V*):

$$Q=TW_{Q},\;K=TW_{K},\;V=TW_{V}$$ (11)

The attention weights and output are computed as:

$${\text{head}}_{k}=\text{Attention}(Q,K,V)=\text{softmax}\left(\frac{QK^{⊤}}{\sqrt{d}}\right)V$$ (12)

The outputs from all heads are concatenated and linearly projected:

$$\text{MHSA}(T)=\text{Concat}({\text{head}}_{1},...,{\text{head}}_{h})W_{O}$$ (13)

This is followed by a feed-forward network (FFN) with GELU activation:

$$\text{FFN}(x)=\text{GELU}(xW_{1}+b_{1})W_{2}+b_{2}$$ (14)

**2) Bidirectional Cross-Attention Mechanism.** After feature refinement, we compute bidirectional cross-attention between support and query sets in both domains. For spatial cross-attention, the support feature *F*_*s*_ and query feature *F*_*q*_ are first linearly projected to form the respective queries, keys, and values:

$$Q_{s}=F_{s}W_{Q}^{s},\;K_{q}=F_{q}W_{K}^{s},\;V_{q}=F_{q}W_{V}^{s}$$ (15)

$$Q_{q}=F_{q}W_{Q}^{q},\;K_{s}=F_{s}W_{K}^{q},\;V_{s}=F_{s}W_{V}^{q}$$ (16)

We then compute two directions of attention:

$$A_{s\leftarrow q}=\text{softmax}\left(\frac{Q_{s}K_{q}^{⊤}}{\sqrt{d}}\right)V_{q},\;A_{q\leftarrow s}=\text{softmax}\left(\frac{Q_{q}K_{s}^{⊤}}{\sqrt{d}}\right)V_{s}$$ (17)

The final spatial comparison feature is obtained by weighted fusion:

$$F_{\text{spatial}}=\lambda A_{s\leftarrow q}+(1-\lambda )A_{q\leftarrow s}$$ (18)

where λ is a learnable parameter that controls the contribution of each direction.

**3) Frequency Domain Cross-Attention.** The frequency-domain comparison is performed using the same structure as above. Let $F_{s}^{f}$ and $F_{q}^{f}$ denote the support and query features in the frequency domain. After linear projection, we compute bidirectional attention:

$$A_{s\leftarrow q}^{f}=\text{softmax}\left(\frac{Q_{s}^{f}(K_{q}^{f}{)}^{⊤}}{\sqrt{d}}\right)V_{q}^{f},\;A_{q\leftarrow s}^{f}=\text{softmax}\left(\frac{Q_{q}^{f}(K_{s}^{f}{)}^{⊤}}{\sqrt{d}}\right)V_{s}^{f}$$ (19)

Then the fused frequency-domain comparison feature is:

$$F_{\text{frequency}}=\lambda_{f}A_{s\leftarrow q}^{f}+(1-\lambda_{f})A_{q\leftarrow s}^{f}$$ (20)

The dual-domain attention mechanisms enhance the matching robustness and enable the model to align task-specific semantics between support and query sets from multiple perspectives.

### 3.7 Feature fusion and classification

To fully leverage the complementary advantages of spatial and frequency domain comparison features, we introduce an attention-based fusion mechanism that adaptively integrates information from both domains. This enhances the discriminative power of the model under few-shot conditions by allowing it to focus on the most informative aspects of the traffic representation.

Let $F_{\text{spatial}}\in {\mathbb{R}}^{L\times d}$ and $F_{\text{frequency}}\in {\mathbb{R}}^{L\times d}$ denote the feature representations obtained from the bidirectional cross-attention modules in the spatial and frequency domains, respectively. Given that both features originate from the same input data and are aligned in dimensionality, we concatenate them along the token axis:

$$F_{\text{concat}}=\text{Concat}(F_{\text{spatial}},F_{\text{frequency}})\in {\mathbb{R}}^{2L\times d}$$ (21)

Next, we apply a Transformer encoder to perform self-attention across the concatenated tokens. This allows the model to explore interactions between spatial and frequency features and learn global dependencies:

$$F_{\text{fused}}=\text{Transformer}(F_{\text{concat}})$$ (22)

Through this attention mechanism, the model adaptively weighs spatial and frequency-domain cues and learns an integrated representation that highlights the most salient features for each task.

**Classification Module.** The fused representation $F_{\text{fused}}$ is then fed into a classification module, implemented as a multi-layer perceptron (MLP), to perform the final intrusion prediction. The classification function is defined as:

$${\hat{y}}_{q}=\text{Softmax}(\text{MLP}(F_{\text{fused}}))$$ (23)

The MLP consists of several fully connected layers with nonlinear activations, such as ReLU or GeLU, to capture complex decision boundaries. The final Softmax layer outputs the probability distribution over *N* classes, enabling the prediction of the query sample’s class label.

By combining spatial and frequency domain features through attention-based fusion, and performing final prediction using a deep nonlinear classifier, the proposed module effectively improves detection accuracy in few-shot scenarios. It captures not only local and domain-specific patterns but also their joint contributions, thereby enhancing robustness and generalization across tasks.

## 4 Experimental results and analysis

### 4.1 Datasets

To evaluate the adaptability and effectiveness of the proposed few-shot intrusion detection method under various real-world conditions, we conduct experiments on two representative benchmark datasets: CICIDS2017 and CICIDS2018. Both datasets were released by the Canadian Institute for Cybersecurity (CIC) and are widely used in intrusion detection research. They simulate realistic network environments and cover a broad range of benign and malicious traffic types [38].

For experimental consistency, we select a subset of attack types from each dataset that contain a sufficient number of samples to construct few-shot tasks. The selected attacks span various intrusion categories, such as Denial-of-Service (DoS), Distributed Denial-of-Service (DDoS), brute-force login attempts, port scanning, and botnet communication. These attack types are commonly encountered in real-world security scenarios and pose diverse detection challenges.

Each few-shot detection task is organized following the *N*-way *K*-shot protocol. That is, *N* different classes (including both benign and attack types) are sampled per task, and each class in the support set contains *K* labeled instances. The query set contains unseen instances from the same *N* classes, which must be classified using information from the support set. This setup closely mimics realistic conditions where labeled attack data are limited and imbalanced.

Table 1 summarizes the attack types used in our experiments along with brief descriptions of their characteristics.

**Table 1:** Attack types and descriptions in CICIDS datasets.

Attack Type	Description
DoS	Single-source flooding to exhaust service resources
DDoS	Multi-source coordinated DoS with increased stealth
FTP-Patator	Brute-force attacks on FTP credentials
SSH-Patator	Brute-force login attempts targeting SSH services
Port Scan	Scanning for open ports to identify vulnerable services
Brute Force	Generic password guessing attacks across protocols
Botnet	Malicious command-and-control traffic from infected hosts

### 4.2 Evaluation metrics

To comprehensively evaluate the performance of the proposed few-shot network intrusion detection method, we adopt five commonly used evaluation metrics: accuracy (ACC), detection rate (DR), precision, false positive rate (FPR), and F-measure (F1-score). These metrics reflect the model’s capability to distinguish between normal and malicious traffic from different perspectives, and collectively assess its practical utility and robustness.

Accuracy measures the overall classification correctness of the model. Detection rate reflects the model’s ability to successfully identify attack samples. Precision describes the proportion of correctly identified attack samples among all samples predicted as attacks. False positive rate measures the proportion of normal traffic misclassified as attacks. F-measure provides a trade-off evaluation between detection rate and precision, especially useful in imbalanced data scenarios.

Let the confusion matrix components be defined as follows:

- *TP*: the number of true positives (i.e., attack samples correctly identified as attacks);
- *TN*: the number of true negatives (i.e., benign samples correctly identified as benign);
- *FP*: the number of false positives (i.e., benign samples incorrectly classified as attacks);
- *FN*: the number of false negatives (i.e., attack samples incorrectly classified as benign).

The above metrics are defined in Eq (24):

$$\left\{ \begin{array}{c} \text{Precision}=\frac{TP}{TP+FP}\\ \text{Detection Rate (DR)}=\frac{TP}{TP+FN}\\ \text{Accuracy (ACC)}=\frac{TP+TN}{TP+TN+FN+FP}\\ \text{False Positive Rate (FPR)}=\frac{FP}{FP+TN}\\ \text{F-measure}=\frac{2\times \text{Precision}\times \text{DR}}{\text{Precision}+\text{DR}} \end{array}\right.$$ (24)

In the following experiments, we evaluate and compare the detection performance of different methods under various task settings using the above metrics, aiming to comprehensively validate the effectiveness and generalization ability of the proposed method.

### 4.3 Experimental setup

The experiments were conducted in the following computing environment: hardware configuration includes an Intel Xeon(R) Platinum 8358P @2.6GHz processor, 64 GB RAM, and an NVIDIA RTX 3090 GPU with 24 GB memory. The software environment uses Ubuntu 22.04 LTS as the operating system, with PyTorch 2.3.0 as the deep learning framework, accelerated by CUDA 12.1 and cuDNN 8.9.

During training, the model adopts an improved Adam optimizer [39], and the relevant hyperparameters are adjusted according to the characteristics of few-shot learning tasks. All experiments are carried out under multiple *N*-way *K*-shot settings to ensure the reliability and representativeness of performance evaluation. The detailed hyperparameter configurations are shown in Table 2.

**Table 2:** Values of hyperparameters.

Hyperparameter	Value
Batch size	32
Number of epochs	300
Learning rate	0.001
Number of classes *N* (CICIDS2017)	5 + 1 (benign)
Number of classes *N* (CICIDS2018)	4 + 1 (benign)
Support set samples *K*	5, 10
Matching module parameter *M*	3
Matching module parameter *L*	3
Matching module parameter *C*	2
MLP layers in classifier	3
MLP hidden units	96

The model is trained using the cross-entropy loss function, which measures the divergence between the predicted class probabilities and the ground truth labels. This loss function is particularly suitable for multi-class classification tasks under few-shot settings, as it effectively penalizes incorrect predictions and encourages the model to assign high confidence to the correct class. During training, the cross-entropy loss is computed over the query set samples in each episode to guide the optimization process.

Formally, given a query sample with ground truth label y∈{1,2,…,N} and predicted probability distribution $\hat{p}=[{\hat{p}}_{1},{\hat{p}}_{2},\ldots ,{\hat{p}}_{N}]$, the cross-entropy loss is defined as:

$${ℒ}_{\text{CE}}=-\log{\hat{p}}_{y}$$ (25)

where ${\hat{p}}_{y}$ denotes the predicted probability assigned to the correct class *y*. The final loss is averaged over all query samples within each episode.

### 4.4 Detection results

To verify the effectiveness of the proposed few-shot intrusion detection method based on multi-domain fusion and cross-attention, we conduct comprehensive experiments on both the CICIDS2017 and CICIDS2018 datasets under two typical few-shot settings: *K* = 5 and *K* = 10.

The experimental results on the CICIDS2017 dataset are shown in Table 3 and Table 4. Table 3 presents the results under the *K* = 5 setting. Despite the very limited number of support samples for each class, the proposed method achieves an average accuracy (ACC) of 97.78%, detection rate (DR) of 97.67%, precision of 97.88%, F-measure of 0.9777, and a relatively low false positive rate (FPR) of 2.12%. Among all classes, the SSH-Patator attack achieves the best detection performance, with an F-measure of 0.9855, indicating the model’s ability to effectively capture SSH-specific attack patterns. Table 4 shows the results for *K* = 10. All metrics are further improved, with average accuracy rising to 99.03%, F-measure reaching 0.9903, and FPR decreasing to just 0.99%. These results demonstrate that increasing the number of support samples, even within a small-sample regime, can significantly improve the model’s matching precision and overall detection performance.

**Table 3:** Detection results on the CICIDS2017 dataset (*K* = 5).

Type	ACC (%)	DR (%)	FPR (%)	Precision (%)	F-measure
DoS	97.07	96.73	2.60	97.38	0.9706
DDoS	97.58	97.47	2.30	97.69	0.9758
FTP-Patator	97.97	98.27	2.33	97.68	0.9797
SSH-Patator	98.55	98.60	1.50	98.50	0.9855
Port Scan	97.72	97.30	1.87	98.12	0.9771
Overall	97.78 ± 0.42	97.67 ± 0.44	2.12 ± 0.39	97.88 ± 0.41	0.9777 ± 0.0030

**Table 4:** Detection results on the CICIDS2017 dataset (*K* = 10).

Type	ACC (%)	DR (%)	FPR (%)	Precision (%)	F-measure
DoS	99.00	99.03	1.03	98.97	0.9900
DDoS	98.80	98.40	0.80	99.19	0.9880
FTP-Patator	99.12	99.47	1.23	98.78	0.9912
SSH-Patator	99.15	99.07	0.77	99.23	0.9915
Port Scan	99.07	99.27	1.13	98.87	0.9907
Overall	99.03 ± 0.29	99.05 ± 0.30	0.99 ± 0.28	99.01 ± 0.27	0.9903 ± 0.0020

The experimental results on the CICIDS2018 dataset are shown in Table 5 and Table 6. Compared with CICIDS2017, the included attack types (Botnet) are more complex, making detection more challenging. Table 5 presents the results under the *K* = 5 setting, where the proposed method achieves an overall accuracy of 97.26% and an F-measure of 0.9726. All metrics confirm the robustness of our model in more complex environments. Table 6 shows the performance under *K* = 10, where the detection metrics are further improved. The overall accuracy rises to 98.64%, the F-measure reaches 0.9864, and the false positive rate is significantly reduced. Notably, the detection improvement for Botnet attacks highlights that the proposed multi-domain fusion and cross-attention mechanism effectively enhances the extraction and discrimination of complex attack behaviors.

**Table 5:** Detection results on the CICIDS2018 dataset (*K* = 5).

Type	ACC (%)	DR (%)	FPR (%)	Precision (%)	F-measure
DoS	97.95	97.90	2.00	98.00	0.9795
DDoS	97.18	97.10	2.73	97.26	0.9718
Brute Force	97.50	96.70	1.70	98.27	0.9748
Botnet	96.42	96.77	3.93	96.09	0.9643
Overall	97.26 ± 0.43	97.12 ± 0.46	2.59 ± 0.40	97.40 ± 0.41	0.9726 ± 0.0031

**Table 6:** Detection results on the CICIDS2018 dataset (*K* = 10).

Type	ACC (%)	DR (%)	FPR (%)	Precision (%)	F-measure
DoS	98.80	98.83	1.23	98.77	0.9880
DDoS	98.73	99.27	1.80	98.22	0.9874
Brute Force	98.70	98.60	1.20	98.80	0.9870
Botnet	98.33	97.97	1.30	98.69	0.9833
Overall	98.64 ± 0.30	98.67 ± 0.31	1.38 ± 0.29	98.62 ± 0.28	0.9864 ± 0.0021

To assess the robustness of the proposed method across different runs, we conducted 10 independent trials for each experimental setting using different random seeds. Considering the significant computational cost of each experiment, we only performed 10 independent runs specifically for the overall results, which are representative of the general performance across classes. Consequently, we report the average and standard deviation (in “mean ± std” format) for the “Overall” rows in Tables 3 to 6. The results show that the F-measure standard deviation is approximately 0.0030 for 5-shot settings and 0.0020 for 10-shot settings, indicating that the proposed method is stable and reliable under few-shot conditions.

In summary, the experimental results show that:

- On both datasets, the proposed method consistently achieves high detection accuracy and low false positive rates, even under few-shot conditions;
- As the number of support samples increases, the model’s ability to distinguish between different attack types improves steadily, indicating that the method can effectively utilize limited information in the support set and has strong discriminative capability for complex attack types.
- The proposed method demonstrates strong stability across independent runs, as evidenced by low standard deviations of overall metrics, suggesting its robustness and practical applicability in real-world few-shot scenarios.

### 4.5 Comparison with related work

To further validate the effectiveness of the proposed few-shot network intrusion detection method, we compare it against several representative recent methods. These methods span diverse strategies, including meta-learning, federated learning, adversarial training, and feature enhancement. The comparison results are presented in Table 7. The evaluation is conducted on both CICIDS2017 and CICIDS2018 datasets under typical few-shot settings with *K* = 5 and *K* = 10, while some works additionally consider extended setups such as *K* = 20 or 1-shot scenarios.

**Table 7:** Comparison of the proposed detection method with related works.

Method/Model (Year)	Dataset	Shots	ACC (%)
FC-Net (2020) [30]	CICIDS2017	5	94.33
FC-Net (2020) [30]	CICIDS2017	10	94.64
Multi-scale deep-CapsNet (2021) [40]	CICIDS2017	5	92.04
Multi-scale deep-CapsNet (2021) [40]	CICIDS2017	10	94.49
Continual learning (2022) [41]	CICIDS2017	5	97.43
Continual learning (2022) [41]	CICIDS2017	10	97.56
FS-IDS (2022) [42]	CICIDS2017	5	97.51
Enhanced triplet network (2022) [43]	CICIDS2017	5	96.18
SPN+K-means (2023) [44]	CICIDS2017	5	94.14
SPN+Masking (2023) [44]	CICIDS2017	5	94.37
SPN+K-means (2023) [44]	CICIDS2017	20	95.63
SPN+Masking (2023) [44]	CICIDS2017	20	96.52
One-shot Siamese network (2023) [45]	CICIDS2017	1	80.81
MetaMRE (2023) [46]	CICIDS2017	10	93.30
MAML+L2F (2023) [47]	CICIDS2018	5	96.00
MAML+L2F (2023) [47]	CICIDS2018	10	97.29
FML+FCN (2024) [48]	CICIDS2017	10	86.07
FML+ResNet (2024) [48]	CICIDS2017	10	87.27
Res-Natural GAN (2024) [49]	CICIDS2018	15	95.75
BS-Agg (2025) [50]	CICIDS2017	N/A	97.90
Self-attention + Iterative refinement (2025) [51]	CICIDS2018	5	96.91
**Proposed method**	CICIDS2017	5	**97.78**
**Proposed method**	CICIDS2017	10	**99.03**
**Proposed method**	CICIDS2018	5	**97.26**
**Proposed method**	CICIDS2018	10	**98.64**

On the CICIDS2017 dataset, the proposed method achieves an accuracy of 97.78% with *K* = 5, outperforming FC-Net (94.33%), Multi-scale deep-CapsNet (92.04%), Enhanced triplet network (96.18%), and SPN+K-means (94.14%). Although SPN-based methods combine unsupervised clustering with meta-learning and perform reasonably well in 5-shot settings, their accuracy remains below 96%. Other methods such as FS-IDS (97.51%) and continual learning method (97.43%) approach our performance but still fall slightly short.

When increasing the support samples to *K* = 10, our method achieves 99.03% accuracy on CICIDS2017, significantly surpassing earlier models such as FC-Net (94.64%) and deep-CapsNet (94.49%), as well as more recent methods like MetaMRE (93.30%) and FML+ResNet (87.27%). Notably, BS-Agg reports an accuracy of 97.90% without a clearly specified *K*, which remains below our result. This demonstrates that the proposed method excels in modeling deep semantic relationships between support and query sets under limited data conditions.

For the CICIDS2018 dataset, relevant studies are comparatively fewer. MAML+L2F achieves 96.00% and 97.29% for *K* = 5 and *K* = 10, respectively, while Self-attention+Iterative refinement method reaches 96.91% under *K* = 5. In comparison, our method achieves 97.26% and 98.64% under the same conditions, reflecting consistent improvement, especially in the 10-shot scenario.

Overall, despite ongoing advancements in network design, feature modeling, and training strategies, many existing methods still rely primarily on single-domain features. They often fail to effectively capture frequency-domain patterns or cross-domain interactions. In contrast, our method leverages a multi-domain fusion strategy and a bidirectional cross-attention mechanism to jointly model spatial and frequency features, allowing deeper comparison between support and query samples. This leads to superior performance under data-scarce conditions.

Furthermore, some methods in Table 7, such as MetaMRE, SPN+Masking, and FML-based methods, focus on alternative tasks such as encrypted traffic classification or continual learning, which are not entirely comparable. However, even under comparable settings, our method consistently achieves superior results, demonstrating strong generalization and applicability across diverse task scenarios.

In summary, the proposed method outperforms all existing methods across multiple few-shot configurations, especially in complex attack environments and low-support conditions, verifying its technical superiority and practical value.

### 4.6 Ablation study

To further validate the effectiveness of each key module in the proposed detection method, we conduct comprehensive ablation experiments on both CICIDS2017 and CICIDS2018 datasets. The objective is to assess the contribution of spatial and frequency domain features to the overall detection performance.

Specifically, we conduct comparisons among the following three model variants:

- **Full Model**: The complete model incorporating both spatial and frequency feature branches, including dual-domain feature extraction and matching;
- **w/o Frequency Feat.**: The model variant with the frequency branch removed; only spatial features are utilized for detection;
- **w/o Spatial Feat.**: The model variant with the spatial branch removed; only frequency features are utilized for detection.

**Fig 6:**
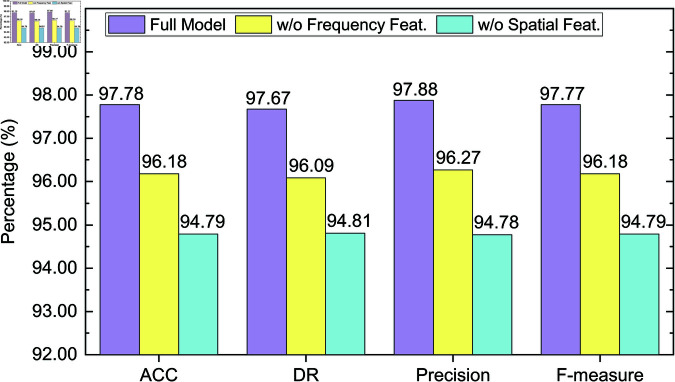
Ablation study results on the CICIDS2017 dataset (K = 5).

**Fig 7:**
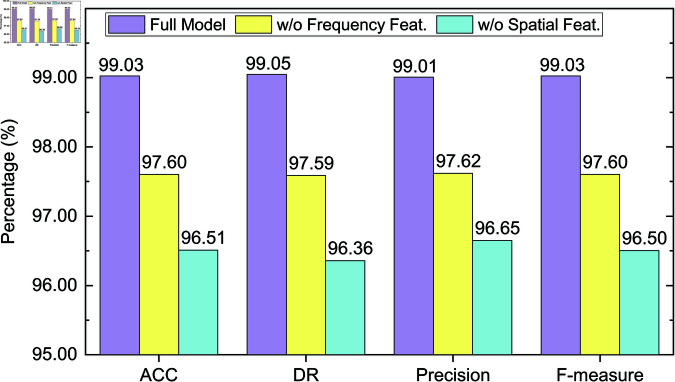
Ablation study results on the CICIDS2017 dataset (K = 10).

**Fig 8:**
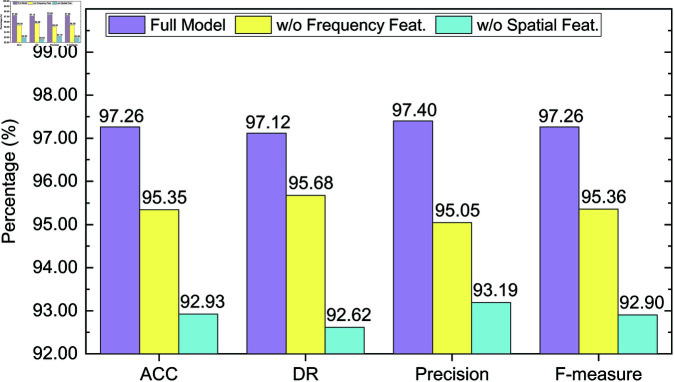
Ablation study results on the CICIDS2018 dataset (K = 5).

**Fig 9:**
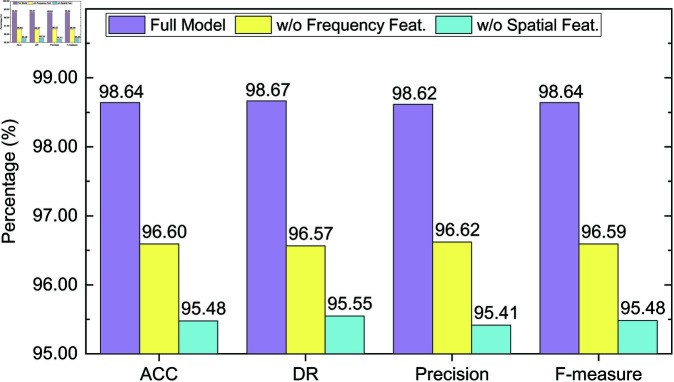
Ablation study results on the CICIDS2018 dataset (K = 10).

Experiments are conducted under two few-shot settings: *K* = 5 and *K* = 10. Evaluation metrics include accuracy (ACC), detection rate (DR), precision, and F-measure. The results are presented in Figs 6, 7, 8 and 9.

On the CICIDS2017 dataset (Figs 6 and 7), the full model achieves high performance under the *K* = 5 setting, with an F-measure of 0.9777. Removing the frequency feature branch reduces the F-measure to 0.9618, indicating the frequency features play an important role in improving detection capability. Further removal of spatial features causes a more significant drop in F-measure to 0.9479, with ACC and DR decreasing to 96.18% and 96.09%, respectively. This demonstrates that spatial features are especially critical in recognizing structured attacks such as DoS and Port Scan. Under the *K* = 10 setting, the full model improves across all metrics, reaching an F-measure of 0.9903. Excluding frequency features results in a drop to 0.9760, and excluding spatial features yields an even lower F-measure of 0.9650. Although the model benefits from increased support samples at *K* = 10, missing domain-specific information still notably degrades performance, validating the necessity of multi-domain fusion.

On the CICIDS2018 dataset (Figs 8 and 9), the full model achieves an F-measure of 0.9726 under the *K* = 5 setting. Removing the frequency branch drops the F-measure to 0.9536, while removing the spatial branch results in a larger decrease to 0.9290. Notably, precision drops most significantly from 97.40% to 93.19%, highlighting the crucial role of spatial features in identifying complex attacks such as Brute Force and Botnet. Under *K* = 10, the full model further improves, achieving an F-measure of 0.9864. Removing the frequency and spatial branches leads to F-measures of 0.9659 and 0.9548, respectively. Despite performance gains with more support samples, the absence of either branch continues to impair detection accuracy, reaffirming the benefit of multi-domain design.

In summary, both spatial and frequency features contribute uniquely to few-shot intrusion detection. Spatial features offer strong discriminative power for structured attacks, while frequency features complement by capturing spectral patterns that are difficult to identify using conventional methods. The synergy between the two domains significantly enhances the model’s robustness and accuracy in sample-scarce environments, confirming the effectiveness and necessity of the proposed multi-domain fusion strategy.

### 4.7 Cross-domain generalization analysis

In real-world cybersecurity applications, network environments and data sources often vary significantly across deployment scenarios. Such variations may include differences in device manufacturers, traffic characteristics, and attack behaviors. To evaluate the adaptability of the proposed method under domain shifts, we conduct cross-dataset experiments to assess its cross-domain generalization capability.

Specifically, cross-domain detection refers to the scenario where the model is trained on a source dataset and directly tested on a target dataset without access to any labeled samples from the target domain. This setting simulates real-world cases where the detection model is deployed in previously unseen or unlabeled environments, making it highly practical.

As shown in Table 8, we train the model on the CICIDS2018 dataset and test it on CICIDS2017 using a 5-way 10-shot setup across all attack types. The model achieves an accuracy of 96.73% and an F-measure of 0.9673, demonstrating strong transferability. Notably, detection performance is best for DDoS and FTP-Patator attacks, with F-measures of 0.9732 and 0.9727 respectively. In contrast, Port Scan exhibits slightly lower performance with an F-measure of 0.9517, due to structural differences in its traffic patterns across datasets. Nonetheless, the results remain acceptable.

**Table 8:** Cross-domain detection results on the CICIDS2017 dataset (*K* = 10).

Type	ACC (%)	DR (%)	FPR (%)	Precision (%)	F-measure
DoS	96.97	97.53	3.60	96.44	0.9698
DDoS	97.32	97.37	2.73	97.27	0.9732
FTP-Patator	97.28	96.63	2.07	97.91	0.9727
SSH-Patator	96.95	96.80	2.90	97.09	0.9695
Port Scan	95.13	95.87	5.60	94.48	0.9517
Overall	96.73 ± 0.39	96.84 ± 0.41	3.38 ± 0.36	96.63 ± 0.38	0.9673 ± 0.0028

Table 9 presents the results of training on CICIDS2017 and testing on CICIDS2018 under the same configuration. The model achieves 95.93% accuracy and an F-measure of 0.9594. While detection for DDoS and Brute Force remains stable with an F-measure of 0.9635, the Botnet class poses greater challenges due to its absence from the training set, yielding a lower F-measure of 0.9515. This suggests that the model learns cross-domain features with strong generalization capability, even for diverse attack types.

**Table 9:** Cross-domain detection results on the CICIDS2018 dataset (*K* = 10).

Type	ACC (%)	DR (%)	FPR (%)	Precision (%)	F-measure
DoS	95.88	96.13	4.37	95.66	0.9589
DDoS	96.37	96.00	3.27	96.71	0.9635
Brute Force	96.32	97.27	4.63	95.45	0.9635
Botnet	95.13	95.53	5.27	94.78	0.9515
Overall	95.93 ± 0.40	96.23 ± 0.42	4.38 ± 0.37	95.64 ± 0.39	0.9594 ± 0.0029

To further examine model performance under lower-resource conditions, we conduct similar experiments under the 5-shot setting, as shown in Tables 10 and 11. The model still achieves competitive results with an overall F-measure of 0.9505 on CICIDS2017 and 0.9424 on CICIDS2018. Additionally, we report standard deviations of key metrics in the Overall rows of each table. The results demonstrate that as the number of support samples increases (from *K* = 5 to 10), not only does the detection accuracy improve, but performance variance also decreases. This confirms the proposed method’s robustness and stability in practical cross-domain few-shot intrusion detection scenarios.

**Table 10:** Cross-domain detection results on the CICIDS2017 dataset (*K* = 5).

Type	ACC (%)	DR (%)	FPR (%)	Precision (%)	F-measure
DoS	95.30	95.23	4.63	95.36	0.9530
DDoS	95.45	95.27	4.37	95.62	0.9544
FTP-Patator	95.22	95.47	5.03	94.99	0.9523
SSH-Patator	95.60	95.67	4.47	95.54	0.9560
Port Scan	93.72	93.43	6.00	93.97	0.9370
Overall	95.06 ± 0.72	95.01 ± 0.76	4.90 ± 0.69	95.10 ± 0.71	0.9505 ± 0.0052

**Table 11:** Cross-domain detection results on the CICIDS2018 dataset (*K* = 5).

Type	ACC (%)	DR (%)	FPR (%)	Precision (%)	F-measure
DoS	94.58	94.93	5.77	94.27	0.9460
DDoS	94.75	94.37	4.87	95.10	0.9473
Brute Force	94.43	94.13	5.27	94.70	0.9442
Botnet	93.22	93.43	7.00	93.03	0.9323
Overall	94.25 ± 0.71	94.22 ± 0.74	5.73 ± 0.68	94.27 ± 0.69	0.9424 ± 0.0051

Overall, although the performance drops slightly compared to within-domain settings, the proposed method still maintains high accuracy and robustness across domains. This confirms that the multi-domain fusion and cross-attention mechanism not only perform well under static conditions but also adapt effectively to dynamic, heterogeneous environments, highlighting the practical value of the method for real-world deployments.

To further demonstrate the superiority of the proposed method in cross-domain generalization, we conduct additional comparative experiments with several traditional baseline models under the same cross-domain settings. Specifically, we evaluate Support Vector Machine (SVM), *k*-Nearest Neighbors (*k*-NN), and Decision Tree (DT) classifiers, as well as a non-attentive deep learning baseline (FC-Net), using the same 10-shot configuration across CICIDS2017 and CICIDS2018.

All baseline models are trained on the source dataset and directly tested on the target dataset without any fine-tuning. The results, shown in Table 12, indicate that while traditional methods achieve acceptable performance in within-domain settings, their effectiveness drops considerably under domain shifts. In contrast, the proposed method achieves superior performance across all key metrics. Specifically, our model reaches an accuracy of 95.93% and an F-measure of 0.9594, outperforming the best traditional baseline (FC-Net) by over 2% in both metrics, and surpassing SVM and *k*-NN by over 10% in F-measure.

**Table 12:** Cross-domain comparison with traditional methods (Train: CICIDS2017, Test: CICIDS2018, overall).

Method	ACC (%)	DR (%)	FPR (%)	Precision (%)	F-measure
SVM	84.07	84.35	16.21	83.88	0.8412
*k*-NN	83.98	83.44	15.49	84.34	0.8389
DT	87.25	87.27	12.77	87.24	0.8725
FC-Net [30]	93.92	94.16	6.32	93.71	0.9394
**Ours**	**95.93**	**96.23**	**4.38**	**95.64**	**0.9594**

These findings further validate that the integration of multi-domain feature encoding and bidirectional cross-attention substantially improves the model’s ability to learn transferable representations, thus enhancing its robustness and practical utility in real-world, heterogeneous network environments.

### 4.8 Model complexity and efficiency analysis

To evaluate the computational efficiency and deployment feasibility of the proposed few-shot intrusion detection framework, we analyze the model in terms of parameter scale, theoretical complexity, and inference performance.

The architecture consists of two structurally identical encoders for spatial and frequency domains, a Mamba-based sequence modeling module, bidirectional cross-attention mechanisms, and a multi-layer perceptron classifier. The total number of trainable parameters is approximately 8.1 million. Among these, the dual-branch encoders contribute about 3.2 million parameters, the Mamba feature extraction module accounts for approximately 2.7 million parameters, the bidirectional cross-attention and domain fusion modules introduce around 1.4 million parameters, and the classification module, implemented as a three-layer MLP, adds approximately 0.8 million parameters. This relatively compact parameter size, compared to large-scale Transformer-based models, demonstrates the lightweight nature of the design, making it feasible for training and inference on moderate hardware resources.

From a theoretical perspective, the use of the Mamba encoder replaces traditional self-attention with a state space modeling approach that achieves linear time complexity 𝒪(n) with respect to sequence length *n*, thereby offering improved scalability and efficiency when processing long network traffic sequences. The bidirectional cross-attention mechanism performs attention operations in both spatial and frequency domains, aligning support and query features with a complexity of $𝒪(d\cdot L^{2})$, where *L* is the token length and *d* is the feature dimension. Due to the stride-based partitioning and downsampling strategy used in the encoder, *L* remains moderate, and the overall attention cost remains manageable. The attention-based fusion module introduces a shallow Transformer to refine the concatenated domain features. Since this module operates over a limited number of tokens, its overhead is minor relative to the total cost.

To assess real-world efficiency, we measure inference time under the 2-way 10-shot setting on an NVIDIA RTX 3090 GPU. The average inference latency per task is approximately 43.2 milliseconds, with memory consumption remaining under 8 GB. These results show that, despite incorporating dual-domain processing and complex attention mechanisms, the proposed model maintains efficient runtime characteristics suitable for real-time deployment.

In summary, the proposed method achieves a balanced trade-off between expressive modeling capability and computational cost. Its modular structure, reduced parameter count, and linear-time encoding strategy enable it to operate efficiently while retaining high detection accuracy, making it practical for applications in dynamic network environments.

### 4.9 Hyperparameter sensitivity analysis

To evaluate the robustness and adaptability of the proposed method under varying few-shot configurations, we conduct a hyperparameter sensitivity analysis focusing on two key parameters: the number of shots (*K*) and the number of classes (*N*) per task. These two parameters directly affect the complexity of the few-shot learning task and are crucial for understanding how the model scales in different scenarios. All experiments in this section are conducted on the CICIDS2018 dataset to ensure consistent and controlled evaluation.

**Fig 10:**
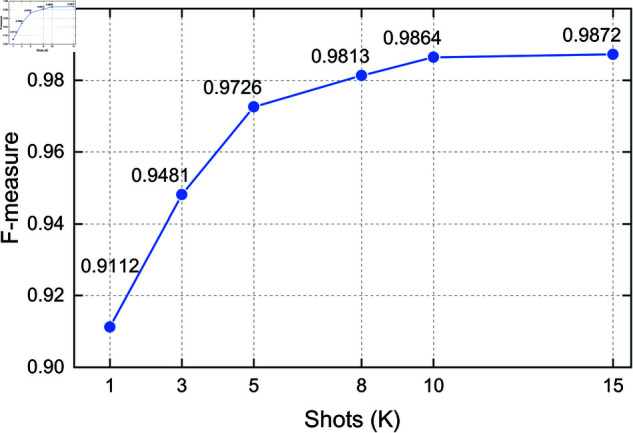
F-measure under varying shot numbers K in 5-way tasks (CICIDS2018).

**Effect of Shot Number (*K*).** We evaluate the model’s performance with *K* values of 1, 3, 5, 8, 10, and 15 in a fixed 5-way classification setting. As shown in Fig 10, the model’s F-measure increases steadily with more support samples per class. Specifically, the F-measure rises from 0.9112 at 1-shot to 0.9872 at 15-shot, showing that the model effectively learns from a small number of examples and stabilizes when *K* exceeds 10.

**Fig 11:**
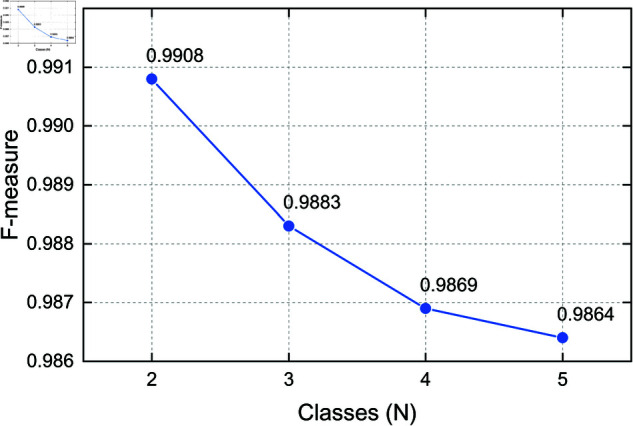
F-measure under varying class numbers N with *K* = 10 (CICIDS2018).

**Effect of Class Number (*N*).** We also examine how the model performs under different *N*-way configurations (2, 3, 4, and 5 classes) with a fixed shot number of *K* = 10. As presented in Fig 11, the model maintains high performance even as the number of classes increases. The F-measure decreases slightly from 0.9908 at 2-way to 0.9864 at 5-way, indicating a graceful performance drop as task complexity increases.

These results confirm that the proposed model is not only effective but also robust across a range of few-shot learning configurations, making it suitable for practical applications where the availability of labeled support data and task complexity can vary.

## 5 Conclusion

To address the challenges of data scarcity and distribution shifts in network intrusion detection, we propose a few-shot detection method based on multi-domain fusion and cross-attention mechanisms. The proposed method jointly models spatial and frequency domain features, enhancing the separability of attack patterns through two-dimensional discrete cosine transform (2D-DCT), and introduces a bi-directional dual-domain cross-attention mechanism to improve the dynamic alignment between the support and query sets. Furthermore, an improved Mamba-based architecture is employed to encode traffic sequences using a selective state space model, effectively capturing long-range dependencies and boosting the model’s capability in complex behavior recognition. Experimental results on CICIDS2017 and CICIDS2018 datasets show that the proposed method achieves F-measures of 0.9777 and 0.9903 (5-shot and 10-shot) on CICIDS2017, and 0.9726 and 0.9864 on CICIDS2018, respectively. Cross-domain experiments further demonstrate its robustness and generalization ability under distributional shifts, with accuracies consistently exceeding 95.13%. These results validate the effectiveness of the proposed method in few-shot settings and its strong potential for real-world deployment. Future work will explore the integration of continual learning mechanisms to enhance the model’s adaptability and update capability in dynamic traffic environments.
